# Functional Liver Recovery After Major Hepatectomy: Integrating Hemodynamic Optimization and Oxidative Stress

**DOI:** 10.3390/jcm15093494

**Published:** 2026-05-02

**Authors:** Vanja Silić, Ivan Romić, Daniela Bandić Pavlović, Goran Pavlek, Gzim Redžepi, Emil Kinda

**Affiliations:** 1 Department of Anesthesiology and Intensive Care, University Hospital Center Zagreb, 10000 Zagreb, Croatia; vanja.silic@gmail.com (V.S.); daniela.bandic.pavlovic@mef.hr (D.B.P.); emil.kinda@gmail.com (E.K.); 2 Department of Surgery, University Hospital Center Zagreb, 10000 Zagreb, Croatia; goranpavlek@gmail.com; 3 Special Hospital Primamed, 10000 Zagreb, Croatia; ravnatelj@primamed.hr

**Keywords:** future liver remnant, functional recovery, oxidative stress, ICG-PDR, LiMAx, SPECT/CT, major hepatectomy

## Abstract

**Background**: Major liver resections that include the removal of four or more Couinaud segments require precise assessment of the future liver remnant (FLR) to prevent post-hepatectomy liver failure (PHLF). Volumetry, although standard in surgical planning, does not always reflect true functional reserve, especially in steatotic, fibrotic or chemotherapy-damaged liver. **Methods**: This review proposes an integrative physiological framework of functional liver recovery after a major hepatectomy that connects preoperative functional assessments—indocyanine clearance (ICG-PDR), Liver Maximum Function Capacity test (LiMAx) and 99mTc-mebrofenin SPECT/CT—with perioperative hemodynamic, oxidative and metabolic parameters. A narrative literature review was performed using PubMed and Web of Science, covering publications from January 2000 to January 2025. The search combined keywords and MeSH terms such as major hepatectomy, liver regeneration, hemodynamic optimization, oxidative stress and post-hepatectomy liver failure. We focused on clinically relevant studies, including randomized controlled studies and consensus guidelines, and complemented the search by screening the reference lists of selected articles. When direct clinical evidence was limited, a physiologically grounded interpretation was used to support a pragmatic framework for perioperative management. **Results**: The framework integrates three complementary physiological domains that together determine functional liver recovery. The first relates to hemodynamic stability, including optimal maintenance of arterial and venous pressures as well as portal-splanchnic gradients, which support adequate perfusion and oxygenation of hepatocytes. The second addresses the balance between oxidative stress and antioxidant defense, where the key indicators are the level of lipid peroxidation and endogenous antioxidant capacity. The third domain evaluates the functional ability of the liver through dynamic tests of synthesis and metabolism, such as factor V, indocyanine clearance (ICG-PDR), and the LiMAx test. With the integration of these three domains, a functional profile of liver recovery can be defined, facilitating monitoring of the physiological response in real time and guiding individualized perioperative support to the individual needs of the patient. **Conclusions**: Functional recovery follows a dynamic continuum, progressing from early reperfusion stress through hemodynamic stabilization to progressive hepatocellular regeneration. Integration of functional FLR assessment with perioperative physiological monitoring may support more individualized prediction of the regenerative capacity and therapeutic decision-making. This physiology-guided perspective extends assessment beyond remnant volume alone to include functional recovery.

## 1. Introduction

Major liver resections require precise assessment of the remaining functional capacity in order to prevent post-hepatectomy liver failure (PHLF). Traditionally, risk was assessed based on volumetry of the future liver remnant (FLR) [1,2]. However, this approach may not reliably reflect functional capacity, particularly in diseased livers. Clinical experience and recent studies have shown that volumetrically adequate FLR does not necessarily guarantee functional stability, especially in patients with steatotic, fibrotic, or chemotherapy-injured liver [3,4,5,6,7]. This discrepancy between volume and true functional capacity has encouraged the development of functional methods of assessment and physiologically based models that connect perfusion, oxidative and metabolic parameters. The aim of this paper is to present an integrative physiological framework of functional liver recovery after major hepatectomy, which integrates hemodynamic stability, oxidative balance and regenerative activity of hepatocytes into a structured conceptual framework.

The literature was identified through searches of the PubMed and Web of Science databases. Studies addressing functional liver reserve, hemodynamic regulation, oxidative stress, and mechanisms of liver regeneration after major hepatectomy were considered. Priority was given to clinical publications relevant to perioperative physiology and the development of post-hepatectomy liver failure.

Where direct clinical evidence is limited, proposed targets are best viewed as physiologically informed hypotheses rather than established standards of care and should be interpreted within the context of clinical judgment.

## 2. Functional Assessment of the Future Liver Remnant

Preoperative evaluation of FLR is key for safe planning of major resections [1,8]. Standard safety thresholds have traditionally been based on volumetry, where in a healthy liver, an FLR of at least 20–25% of total volume is commonly considered acceptable in clinical practice. In patients with steatosis or damage after chemotherapy, a minimum of 30–40% is recommended, while in cirrhosis and portal hypertension, a volume greater than 40–50% is generally considered necessary [6,9]. However, these thresholds do not reflect the true functional ability of the remaining parenchyma [3,4,9]. Functional capacity may be substantially reduced despite apparently adequate volume, especially in the presence of heterogeneous or impaired perfusion at the microvascular level, steatosis, or reduced metabolic reserve. This has contributed to the adoption of the concept of functional FLR. This approach complements volumetric analysis with dynamic functional testing. The most commonly used methods include indocyanine green clearance (ICG-PDR) and retention of dye after 15 min (R15) [10,11], which represent a global measure of the excretory function of the liver. Values ICG-PDR < 10%/min or R15 > 20% have been associated with a high risk of PHLF development [10,11]. The LiMAx test (Liver Maximum Function Capacity) evaluates cytochrome P450 (CYP1A2) enzymatic activity, and preoperative values < 315 μg/kg/h have been associated with increased risk of postoperative complications [3,11]. In addition, 99mTc-mebrofenin SPECT/CT allows regional functional mapping of the liver, combining anatomical and functional data. This supports decision-making regarding the need for additional growth stimulation procedures such as portal vein embolization (PVE), associating liver partition and portal vein ligation for staged hepatectomy (ALPPS) or liver venous deprivation (LVD) [12,13]. Liver venous deprivation is defined as the combined embolization of portal and hepatic veins to induce hypertrophy of the future remnant. With the integration of these methods it is possible to precisely determine the real functional capacity of the remaining tissue and to timely recognize patients in whom an additional preoperative growth-stimulating procedure is needed. This approach suggests that volume alone may not be sufficient for risk assessment, and that functional reserve should also be taken into account whenever possible [1,4,14].

## 3. Hemodynamic Determinants of Liver Recovery

### 3.1. Hemodynamic Stability and Microcirculation

Hemodynamic stability and preserved microcirculation are fundamental determinants of functional liver recovery after major resection [2,9,14]. Functional recovery does not depend solely on the volume of remaining parenchyma, but more so on its perfusion, oxygenation and metabolic support in the early hours and days post-operation. Perfusion and congestion present two interconnected physiological processes. Increased portal inflow through a smaller remnant may stimulate regeneration; however, if there is an imbalance in sinusoidal congestion, endothelial dysfunction and hypoxic hepatocytes may develop [14]. Postoperative impairment of liver perfusion may also relate to small-for-size syndrome, where the remnant liver mass is insufficient relative to portal flow. Contrarily, a shortage of perfusion and ischemic parenchyma could retard the regeneration process and increase the risk of post-hepatectomy failure of the liver (PHLF) [2,6]. A key physiological parameter linking portal inflow and venous outflow is portal-splanchnic gradient pressure (PSPG), defined as the difference between portal venous pressure and central venous pressure (CVP). While hepatic venous pressure gradient (HVPG) is a standard measure of portal hypertension, PSPG is introduced here as a complementary physiological concept that may better capture intraoperative hemodynamic balance between portal inflow and venous outflow. The concept of portal-splanchnic pressure gradient (PSPG) is proposed as a physiological framework rather than an established clinical metric. This parameter reflects the balance between perfusion and congestion: too low PSPG values (<5 mmHg) indicate insufficient portal flow and risk of ischemic injury, while elevated PSPG values (>8–10 mmHg) have been associated with sinusoidal congestion, edema and increased risk of PHLF [9,15]. A PSPG range between 5 and 8 mmHg may be physiologically favorable in a selected context. This has not been prospectively validated. PSPG monitoring may be considered (via catheterization of the mesenteric circulation and central venous system) or non-invasively by Doppler ultrasound combined with CVP during the first 12–24 h after surgery, when the liver undergoes a phase of hemodynamic adaptation [2,13].

**Figure 1:**
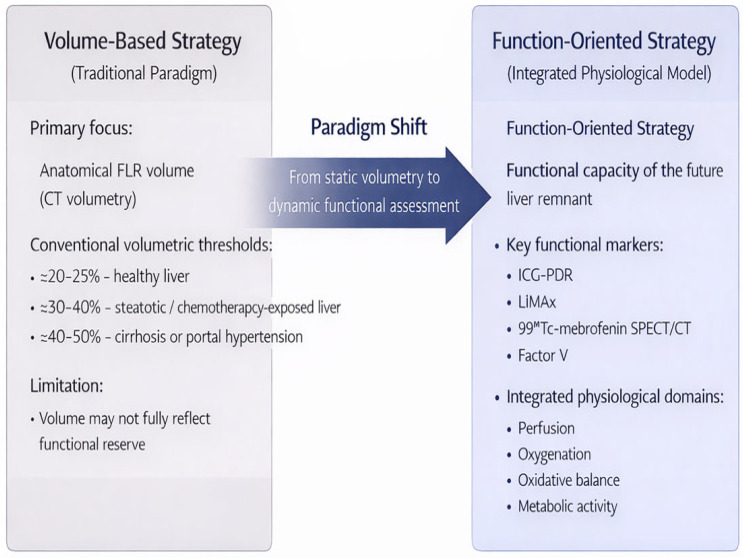
Conceptual shift from volumetric to function-based assessment of the future liver remnant (FLR).

This hemodynamic perspective illustrates the broader paradigm shift from purely anatomical FLR thresholds toward an integrated function-based approach to liver recovery (Figure 1).

### 3.2. Hemodynamic Goals and Anesthetic Management

In the intraoperative and early postoperative period, the aim is to maintain an appropriate balance between perfusion and bleeding risk [2,3,15]. Too high central venous pressure (CVP) increases blood loss and congestion of the liver, while excessively low CVP may compromise portal perfusion [2,15]. An individualized approach should include titration of fluid and vasopressor therapy according to proposed perfusion targets of mean arterial pressure (MAP) 70–80 mmHg, central venous pressure (CVP) < 5 mmHg, and PSPG 5–8 mmHg, recognizing that these ranges are derived from physiological and observational data [15]. Such a personalized strategy may help maintain balance between flow, oxygenation and venous drainage. In many centers, a relatively restrictive fluid strategy is adopted perioperatively, often combined with albumin administration to maintain oncotic pressure while avoiding volume overload [13]. In cases of elevated PSPG, strategies to reduce venous congestion, such as ultrafiltration, may be considered [9,15]. Before considering ultrafiltration, diuretic therapy may be used to optimize intravascular volume status, particularly in the absence of significant hemodynamic instability. Excessive doses of adrenaline and dopamine should be avoided as they may exacerbate sinusoidal stress. The key parameters are summarized in Table 1.

## 4. Oxidative Stress and Redox Regulation

### 4.1. Pathophysiology of Oxidative Stress

Reperfusion injury of the liver is considered one of the key factors influencing functional recovery after major liver resection [8,12,14]. Ischemia–reperfusion injury, particularly in the context of the Pringle maneuver or total vascular exclusion, is a major contributor to oxidative stress. Although re-establishing flow through the remaining parenchyma is necessary for hepatocyte survival, the moment of reperfusion may trigger a sudden rise in the production of reactive oxygen species (ROS) and consequent cell damage [8,12]. Intermittent portal clamping may reduce oxidative stress through ischemic preconditioning. During ischemia, reserves of ATP are exhausted and accumulation of hypoxanthine in hepatocytes can occur. At the moment of reperfusion, a sudden influx of oxygen can activate xanthine oxidase and stimulate production of superoxide anions (O_2_^−^), hydrogen peroxide (H_2_O_2_) and hydroxyl radicals (OH). These reactive species attack the membrane, proteins and DNA, causing lipid peroxidation and damage to the cell [8,12]. One of the most commonly used markers of lipid peroxidation is malondialdehyde (MDA), whose elevated plasma levels reflect the peak of reperfusion stress [8,14]. Simultaneously, the antioxidative system (prevalently glutathione (GSH) and superoxide-dismutase (SOD)) acts as a key defense mechanism that neutralizes free radicals and sustains redox balance [7,14]. These biomarkers are not yet standardized for routine clinical use and are primarily used in a research setting, with limited integration into clinical decision-making. Thus, their role in perioperative management remains supportive rather than directive. When this balance is disturbed, oxidative imbalance occurs, which may lead to mitochondrial dysfunction, inflammatory activation and hepatocellular necrosis [8,12].

### 4.2. Dynamic Balance Between Oxidative and Regenerative Response

In the first hours after resection, the oxidative phase dominates, marked by an increase in MDA and a decrease in antioxidant reserves. During the next 24–48 h, with activation of regenerative mechanisms, levels of GSH and SOD gradually rise, while MDA decreases, marking the transition from reperfusion to the regenerative phase [7,14].

**Figure 2:**
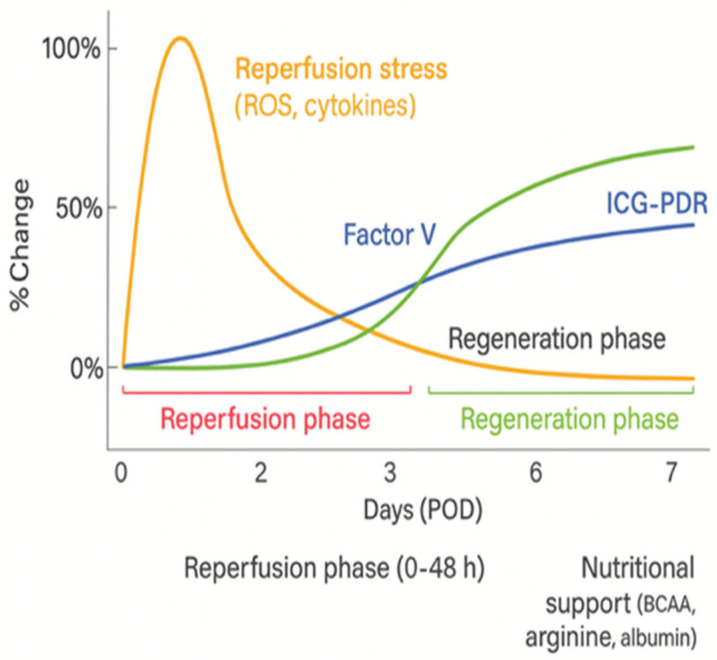
Temporal dynamics of oxidative stress and antioxidant defense after hepatectomy.

This dynamic change is shown in Figure 2 which illustrates the physiological shift from oxidative imbalance toward restored redox homeostasis.

### 4.3. Surgical and Pharmacologic Strategies for Reperfusion Control

Limiting the oxidative surge after reperfusion is considered an important goal in hepatobiliary surgery. To prevent a sudden oxidative load, controlled reperfusion techniques may be used to enable the gradual return of blood into the remaining parenchyma. One commonly described approach is the so-called “slow unclamping” technique, in which the clamp on the portal vein and hepatic artery is released gradually—first 25%, then 50%, 75% and finally 100% within 1–2 min [9,12]. Such gradual reperfusion may allow the liver to adapt to the increase in flow and thereby avoid sudden oxidative stress. Additionally, in some centers flush reperfusion is used, involving rinsing of liver parenchyma before reperfusion with a cold solution (Ringer lactate with albumin) at moderate hypothermia of 33–35 °C, whereby ischemic metabolites may be removed and mitochondrial function potentially stabilized [12].

### 4.4. Metabolic and Antioxidant Support

Prevention of oxidative injury is not based only on surgical techniques, but also on pharmacological and metabolic protection. Antioxidant strategies may also include vitamins (C and E), selenium, N-acetylcysteine, and coenzyme Q10. Their clinical benefit in this setting remains incompletely established. N-acetylcysteine (NAC) is frequently discussed as a central component of this approach which may increase endogenous antioxidant capacity of hepatocytes and decrease the level of malondialdehyde (MDA) [8,14]. N-acetylcysteine (NAC), administered perioperatively, replenishes intracellular glutathione stores and attenuates lipid peroxidation, as reflected by reduced MDA levels. In addition to NAC, prostaglandin E_1_ (PGE_1_) and prostacyclin (iloprost) are commonly incorporated to enhance microcirculation, decrease sinusoidal vasoconstriction and inhibit aggregation of thrombocytes, and in combination with NAC have been reported to demonstrate a synergistic effect in decreasing the formation of reactive oxygen species (ROS) and proinflammatory cytokines like TNF-α and IL-6 [7,9,12]. Additional support may be provided by albumin, which, besides volume effect, also has antioxidant and endothelial-stabilizing properties [8,14], and in combination with prostacyclin may further improve oxygenation of the microcirculation [9,12]. Considering that prostaglandins can cause mild systemic hypotension, they are frequently combined with low doses of noradrenaline to maintain stable mean arterial pressure (MAP 70–80 mmHg) [13,15]. This reflects an important trade-off, as attempts to improve microcirculation may at the same time compromise systemic hemodynamic stability.

### 4.5. Biomarkers of Oxidative Stress

Clinical indicators of oxidative imbalance include increases in transaminases, lactate and bilirubin. Specific biochemical markers such as MDA, GSH/GSSG ratio, activity of superoxide dismutase (SOD) and inflammatory mediators such as IL-6 and TNF-α allow more precise assessment of the metabolic stress of the liver [8,12]. Elevated MDA levels together with decreased GSH and SOD indicate dominance of oxidative processes, while their gradual increase within 48 h marks the transition from oxidative phase to the beginning of functional regeneration [14,16].

## 5. Functional Markers of Liver Recovery

Assessment of the functional capacity of the liver after major resection plays an important role in early recognition of patients at risk for post-hepatectomy liver failure (PHLF). While biochemical tests reflect consequences of injury, functional tests may provide dynamic insight into the ability of the remaining parenchyma for synthesis, metabolism and clearance of substances [1,3,11]. This distinction between “indicators of injury” forms the basis of current approaches to assessment of regenerative potential.

### 5.1. Biochemical Tests: Indicators of Hepatocellular Injury

Biochemical tests constitute the first step in evaluating liver function, yet do not give direct information on actual functional capacity [1,17]. Direct bilirubin more accurately reflects biliary excretory function and biliary patency, while total bilirubin may be influenced by multiple factors, including hemolysis and hepatocellular dysfunction. INR/prothrombin time (PT) demonstrates the capability for synthesis of coagulation factors, but can be affected by the influence of vitamin K and transfusion interventions and may therefore limit specificity [6,10]. AST and ALT reflect acute hepatocellular injury, where their rapid decline in the first postoperative days has positive prognostic significance, while persistent elevation suggests persistent hepatocellular injury or reperfusion stress [8,12]. Recently, the AST-to-platelets ratio index/albumin-to-bilirubin index (APRI/ALBI score) was suggested to be used as a simple and dynamic liver function recovery monitoring method after the first ALPPS stage [18].

Biochemical parameters are useful for daily monitoring, but do not allow prediction of regenerative potential or quantification of functional capacity in real time [1,3].

### 5.2. Functional Tests: Dynamic Assessment of Hepatic Capacity

Unlike biochemical tests, functional tests can allow real-time evaluation of the ability of the liver to synthesize and metabolize substances, and are therefore important for early identification of patients at risk of PHLF [3,4,11]. Among the most important indicators are factor V, indocyanine clearance (ICG-PDR) and the LiMAx test (Liver Maximum Function Capacity). Additional functional assessments may include MELD-based evaluation and other dynamic liver function tests, depending on the clinical context. Factor V is independent of vitamin K status and is considered a sensitive early indicator of protein synthesis [6,10]. An increase in concentration within 24 h after surgery may indicate successful regeneration, while values lower than 30% are associated with increased risk of PHLF.

Indocyanine clearance (ICG-PDR) measures the rate of elimination of dye that is almost exclusively cleared by the liver. Values lower than 10%/min may reflect reduced perfusion and functional capacity, while their rise in early postoperative days indicates good recovery [11]. The LiMAx test quantifies cytochrome P450 (CYP1A2) enzymatic activity after intravenous administration of methacetin labeled with ^13^C. The results are expressed in μg/kg/h, and values lower than 150 indicate a high risk of PHLF [3]. Unlike conventional biochemical tests, LiMAx may allow earlier detection of reduced functional capacity even before the appearance of laboratory abnormalities. A further approach that has been explored is systemic caffeine clearance, which reflects hepatic metabolic capacity through cytochrome P450 1A2 activity. Although it has shown good sensitivity in detecting changes in liver function, its use has largely remained confined to research settings. In clinical practice, its application is limited by methodological complexity and the absence of standardized protocols [17]. 

With the integration of these indicators it is possible to define a functional profile of regeneration, which reflects in real time the ability of the liver to synthesize proteins, elimination of toxins and metabolic stability [18,19,20]. Such an approach may enable timely adjustment of therapeutic and nutritional interventions and personalized monitoring of patients in the early postoperative period.

### 5.3. Dynamics of Functional Recovery

During the first 48 h after major resection, the transition between reperfusion stress and the beginning of functional regeneration occurs [7,12]. Successful regeneration is typically accompanied by a gradual increase in factor V, improvement in ICG-PDR values and decrease in lactate concentration with stabilization of hemodynamic status [3,10,11]. At the same time, biochemical tests show a decrease in bilirubin and transaminases, which may reflect the transition from catabolic to regenerative phase [6]. Integrated assessment of these parameters may facilitate early recognition of patients who need additional hemodynamic, metabolic or nutritional support [21,22]. With a combination of biochemical and functional indicators, it may be possible to distinguish morphological recovery from true functional stabilization of the liver. In this context, factor V and ICG-PDR may be particularly useful for early therapeutic decision-making in the intensive care unit—for example, in planning fluid management, nutritional support or continuation of antioxidant therapy (NAC, prostacyclin). In the integrated model of monitoring, biochemical tests reflect past injury, while functional tests show what the liver can still achieve. This distinction has direct implications for clinical decision-making in the early postoperative period.

## 6. Integrated Model of Liver Recovery

### 6.1. The Molecular Level

**Figure 3:**
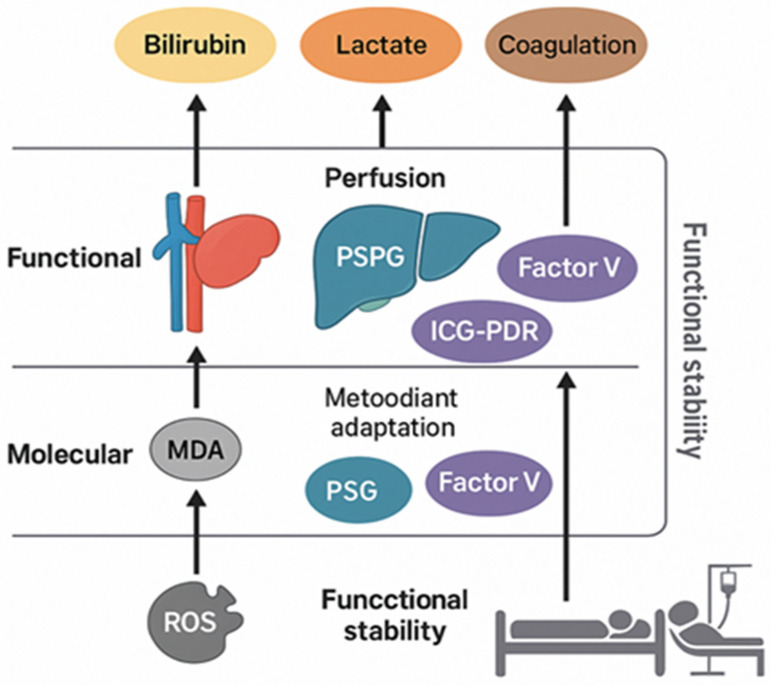
Integrated “molecule-to-bedside” model of functional liver recovery after major hepatectomy.

Recovery of the liver after major resection is a complex, multilayered process that includes the interaction of molecular, microcirculatory and clinical mechanisms [7,22]. An integrated approach, which connects oxidative stress, perfusion balance and functional indicators, may enable a more complete understanding of the underlying dynamics of liver regeneration and help prevent post-hepatectomy liver failure (PHLF) [10]. Regeneration of the liver after a major resection begins within the first hours after surgery and takes place through three interconnected levels: molecular, functional and clinical. The interaction between oxidative stress, microcirculatory adaptation and dynamic functional biomarkers can be viewed as a continuum from cellular response to bedside management. This integrated “molecule-to-bedside” framework is illustrated in Figure 3.

At the molecular level, regeneration is initiated by activation of Kupffer cell and sinusoidal endothelial cells which release cytokines such as TNF-α, IL-6 and HGF, thereby promoting the transition of hepatocytes from the resting phase (G_0_) to growth phase (G_1_) [1,2]. At the same time, reperfusion is associated with the generation of reactive oxygen species (ROS), which temporarily disrupts redox balance [3,4]. The establishment of antioxidant defense, characterized by increased activity of glutathione (GSH) and superoxide dismutase (SOD), marks a transition from the oxidative phase toward regenerative recovery [3,4,5]. If ROS production exceeds antioxidant defense mechanisms, mitochondrial dysfunction and endothelial damage may occur, which may impair hepatocyte proliferation. Thus, control of oxidative stress is an important determinant of successful regeneration and preservation of microcirculation [4,5].

### 6.2. The Functional Level

At the functional level, recovery is closely linked to balanced hemodynamics. Perfusion needs to be sufficient to ensure adequate oxygenation of hepatocytes, but not excessive, so as to avoid congestion and sinusoidal edema [6,7,8]. Optimal portal-splanchnic gradient (PSPG) values typically range between 5 and 8 mmHg, with CVP < 5 mmHg and mean arterial pressure (MAP) of 70–80 mmHg. Maintaining these parameters may support adequate microcirculation and minimize oxidative stress, thereby creating conditions for a successful regeneration of liver parenchyma. At the same time three important processes occur: renewal of flow and sinusoidal oxidation, reorganization of the vascular net and reactivation of hepatocellular synthesis and detoxification of the mechanism. Early establishment of hemodynamic balance appears to be an important factor in stimulating regeneration signals and rejuvenation of liver architecture [7,8].

### 6.3. The Clinical Level

At the clinical level, regeneration is assessed using dynamic functional tests and hemodynamic indicators. An increase in factor V and ICG-PDR during the first 48 h after resection may indicate restoration of synthetic and perfusion function of the liver. At the same time, the decrease in lactate, bilirubin and transaminases may reflect the transition from reperfusion stress to the regenerative phase. Monitoring these parameters in real time may allow early recognition of patients at risk of PHLF and timely adjustment of targeted support—hemodynamic, metabolic or nutritional [9,10,11].

### 6.4. Integrative Concept

The integrated model of liver recovery can be summarized across three interconnected levels—molecular, functional, and clinical—as shown in Table 2.

## 7. Conclusions and Future Directions

The functional recovery of the liver after major resection is not a linear process, but rather the result of a dynamic interaction between hemodynamics, oxidative balance and regenerative mechanisms. The traditional volumetric approach, based solely on the size of the future liver remnant (FLR), may no longer reliably predict clinical outcome. The modern concept places emphasis on the functional capacity of the remaining parenchyma. Successful regeneration requires a fine balance between sufficient perfusion and minimal congestion, with control of reperfusion-related oxidative stress and targeted pharmacological and metabolic protection. Hemodynamic adaptation and preservation of microcirculation may influence activation of antioxidant systems (GSH and SOD), which form the molecular basis of regenerative processes. Functional indicators—above all factor V, ICG-PDR and LiMAx—enable real-time monitoring and early recognition of liver insufficiency, while integration with hemodynamic and oxidative markers provides a framework for personalized perioperative management of patients. This physiological approach redirects the focus of postoperative care from volume to actual functional recovery of the liver. This review proposes an integrative physiological framework for understanding and supporting functional recovery of the liver after major hepatectomy. While previous studies have examined volumetric, metabolic, or hemodynamic factors of liver regeneration separately, this work aims to integrate these processes into a single conceptual framework—the perfusion–oxidative–regenerative continuum. The article outlines physiological targets (PSPG 5–8 mmHg, CVP < 5 mmHg, MAP 70–80 mmHg, DO2 > 500 mL/min/m^2^) and correlates them with oxidative (MDA and GSH/SOD) and functional (Factor V, ICG-PDR, and LiMAx) markers, providing a pragmatic framework for individualized perioperative management. By emphasizing the anesthesiologist’s active role in optimizing perfusion, oxygen delivery, and redox homeostasis, this paper bridges experimental physiology and clinical practice—offering a translational link “from molecule to bedside”.

## Figures and Tables

**Table 1:** Integrated hemodynamic, functional, and oxidative stress-related parameters relevant to liver recovery after major hepatectomy.

Markers	What It Measures	Clinical Availability	Evidence Level	Proposed Threshold/Interpretation	Potential Clinical Implication
PSPG	Balance between portal inflow and hepatic outflow	Not routinely available; no standardized method	Conceptual/emerging	~5–8 mmHg (proposed physiological range, not yet clinically validated)	May support individualized hemodynamic interpretation
MAP	Systemic perfusion pressure	Routinely available	Moderate	≥65 mmHg (minimum target, may require individualization)	Maintenance of organ perfusion
CVP	Venous congestion and hepatic outflow resistance	Routinely available	Moderate	Lower CVP may reduce hepatic congestion, particularly during parenchymal transection	May reduce hepatic congestion
ICG-PDR	Dynamic liver function and perfusion	Available in specialized centers	Moderate–high	>15–18%/min (approximate functional threshold)	Functional assessment of liver recovery
Factor V	Synthetic liver function	Routinely available	Moderate	Decline reflects reduced synthetic capacity and may indicate liver dysfunction	Early indicator of dysfunction
MDA	Lipid peroxidation (oxidative stress)	Research setting	Low	No standardized threshold; elevated levels reflect oxidative injury	Research marker of oxidative injury
GSH/GSSG ratio	Redox balance	Research setting	Low	No standardized threshold; reflects systemic redox balance	Indicator of antioxidant capacity
SOD activity	Antioxidant enzyme activity	Research setting	Low	No standardized threshold; reflects antioxidant response capacity	Reflects oxidative stress response

Abbreviations: PSPG, portal-splanchnic pressure gradient; MAP, mean arterial pressure; CVP, central venous pressure; ICG-PDR, indocyanine green plasma disappearance rate; MDA, malondialdehyde; GSH/GSSG, reduced/oxidized glutathione ratio; SOD, superoxide dismutase.

**Table 2:** Integrated framework of liver recovery after major resection, linking dominant biological processes with measurable recovery indicators at the molecular, functional, and clinical levels.

Level	Dominant Processes	Recovery Indicators
Molecular	Oxidative stress, (ROS), restoration of antioxidant defense (GSH and SOD), activation of IL-6 and TNF-α signaling	Decrease in MDA, increase in GSH and SOD
Functional	Perfusion balance, microcirculatory stability, hepatocyte regeneration	PSPG 5–8 mmHg (reported range), increase in Factor V, ICG-PDR
Clinical	Stabilization of metabolic and synthetic functions	Decrease in bilirubin and lactate, normalization of INR

## Data Availability

No new data were created or analyzed in this study.
